# Current Practices, Willingness and Perceived Ability to Implement an Injury Prevention Exercise Program Among Post‐Primary Physical Education Teachers

**DOI:** 10.1111/josh.13242

**Published:** 2022-08-27

**Authors:** Frank Devereux, Enda Whyte, Johann Issartel, Sarahjane Belton, Siobhan O'Connor

**Affiliations:** ^1^ Department of Science & Health Institute of Technology Carlow Co. Carlow Ireland; ^2^ Centre for Injury Prevention & Performance, School of Health & Human Performance Dublin City University Whitehall Dublin 9 Ireland; ^3^ School of Health & Human Performance Dublin City University Whitehall Dublin 9 Ireland; ^4^ LifePAC Research Centre, School of Health & Human Performance Dublin City University Whitehall Dublin 9 Ireland; ^5^ Centre for Injury Prevention & Performance School of Health & Human Performance Dublin City University Whitehall Dublin 9 Ireland

**Keywords:** injury prevention, post‐primary, school, physical education, PE teachers, implementation

## Abstract

**Background:**

Musculoskeletal injuries are common in adolescents, and recently schools have been suggested as an opportune location for injury prevention strategies. This study aimed to identify the current practices and perceptions of post‐primary PE teachers in Ireland on injury prevention exercise programs (IPEP), which are key to informing potential implementation strategies.

**Methods:**

Post‐primary PE teachers (n = 287) completed an online anonymous survey. Outcome measures included current IPEP practices in PE class, teachers' attitudes toward IPEPs, willingness to implement, and perceived ability to implement an IPEP in PE class. Descriptive statistics were calculated, and Mann‐Whitney *U* tests were used to compare differences between groups.

**Results:**

Results indicated that only 1 in 5 PE teachers currently used an IPEP in class. Of these, no teacher used an existing IPEP exactly as intended, while most teachers were willing to implement an IPEP (80.5%). Those who previously received formal IPEP education or were aware of an existing IPEP had significantly higher perceived ability to implement an IPEP in class (p < .001).

**Conclusions:**

This study demonstrates that despite a willingness among PE teachers to implement IPEPs in class, few currently do. Thus, post‐primary PE class may be an under‐utilized setting for adolescent injury prevention and warrants further investigation.

Worldwide, more than 1 in 3 of the adolescent population suffer from a musculoskeletal injury yearly,^1^, ^2^, ^3^ with up to 35.6% of Irish adolescent males suffering at least one injury in a single academic year.^4^ One of the leading causes of musculoskeletal injuries is previous injury.^5^ Previous lower limb injury increases the risk of sustaining a range of subsequent lower limb injuries.^6^ Thus, early preventative interventions aimed at reducing the risk of sustaining an initial injury may have long‐term benefits for the population as these injuries can result in a physical,^5^ psychological,^7^ and financial burden for adolescents.^8^, ^9^ Injury prevention exercise programs (IPEPs) are programs typically consisting of exercises to improving balance, strength, and agility,^10^ and are a well‐established means reducing injury risk in adolescents in sport.^10^, ^11^, ^12^ However, more recently there have been calls for IPEPs to be implemented within more generalized healthy and active populations to improve its impact on public health.^13^

Recent research has attempted to explore the effects of school‐based IPEPs. Two studies have shown that 12‐week and 23‐week school‐based IPEPs reduced the risk of injury in adolescent females in Canada^14^ and New Zealand,^15^ respectively. This may be explained, in part, by the fact that physical education (PE) class in schools offer a unique opportunity for IPEPs due to compulsory attendance and dedicated timeslots, highly skilled PE teachers', appropriate facilities, and a supportive environment,^16^ overcoming some of the traditional barriers to implementation in sport.^17^ However, this preventative effect was not evident in adolescent males, potentially due to the floor effect of the IPEPs preventative capabilities, whereby the IPEP may not have been sufficiently challenging to stimulate adaptation, due to the higher general base‐level of neuromuscular control in adolescent males.^14^ While results from these 2 studies are promising, further research is needed from other counties to advance these findings.

In Canada, the most significant barriers to implementation of a school‐based IPEP included intervention complexity and readiness for implementation, while facilitators included evidence strength and adaptability of the program. Meanwhile, PE teachers' perceived ability to implement the program, and their willingness to fully engage in program implementation were considered to potentially be either a facilitator or barrier to school‐based IPEP implementation.^18^

Prior to the development of a school‐based IPEP, it is imperative to analyze the components influencing its success. PE teachers have clear insight into many of these factors such as the perceived need for change, readiness for implementation, and participant characteristics (both implementers and target population),^19^ as they liaise with school management, create lesson plans, and engage with students daily. Once determined, future programs can attempt to mitigate any identified barriers and amplify facilitators to enhance implementation. A multistrategy approach that addresses identified barriers can lead to significant increases in implementation and maintenance of physical activity.^20^

As settings change, so too must the implementation strategy.^19^, ^21^ Despite promising evidence from Canada and New Zealand on the efficacy of school‐based IPEPs, knowledge as to what extent IPEPs are currently used in PE classes remains unclear. For programs to be successfully implemented and sustainable in school settings, this information is crucial as it informs the development of a school‐based IPEP. Therefore, the purpose of this study is to establish the current practices, willingness, and perceived abilities to implement an IPEP among post‐primary PE teachers in Ireland.

## METHODS

### Participants

Post‐primary schools in Ireland cater for students from the ages of 12‐18 and is compulsory for students under the age of 16. To be included in this study, post‐primary PE teachers must have taught PE in either of the previous 2 academic years. A recruitment email (n = 723) was sent to every post‐primary school in Ireland which included information about the survey and a link to the survey itself. We requested the school to distribute the survey to all PE teachers in their school. Reminder emails were sent 3 and 6 weeks post‐initial email. Additionally, the survey was advertised via social media, word of mouth and emails to past graduates in 2 accredited PE teaching programs in Ireland.

### Procedure

An anonymous cross‐sectional online survey was used to establish current injury prevention exercise program (IPEP) trends and perceptions among PE teachers in Irish post‐primary schools. This survey was adapted from previous research,^22^ and validated by experts in the fields of injury prevention (n = 2), PE academia (n = 2), and PE teachers (n = 3). Each question was rated between 1 and 5 for clarity, comprehensiveness, and appropriateness, with questions averaging a score of less than 4/5 being modified or removed. In addition, experts were requested to provide recommendations for question and survey improvement.^23^ Once validated, the survey was piloted with 12 eligible PE teachers. The survey took on average 10:03 minutes (±5:17 minutes) to complete. Sample size calculations recommended a minimum sample size of 228 respondents (5% margin of error, 95% CI). The survey was open for response collection from April 12, 2021 to June 14, 2021. The survey was administered online using Qualtrics (SAP America Inc., Seattle, WA).

### Instrument

The survey included 6 sections and a total of 33 questions (supplementary material 1). For the purposes of this survey, 2 definitions were provided for respondents at the beginning of the survey, which were developed by the research team. Injury prevention exercises were defined as “physical activities performed with the aim of reducing the risk of sustaining an injury.” Injury prevention exercise program (IPEP) was defined as “a group of injury prevention exercises performed with the aim of reducing the risk of injury and can also be used to increase the persons preparedness for future activity.”

Section 1 consisted of 12 demographic questions. Participants were asked their gender, number of years they have been teaching PE, which year groups they currently taught, and if they had any formal IPEP education. Additionally, details relating to the school they were teaching in were collected. Section 2 (2 questions) examined participants' awareness of IPEP programs. In section 3 (10 questions), their recent use of an IPEP in PE class was queried. If they answered “yes,” teachers were requested to provide information on what encouraged them to perform an IPEP, where they sourced it, and detail the IPEP components, frequency, and duration. Section 4 (2 questions) assessed each participants' level of agreement for 11 statements regarding their current attitudes toward IPEPs. A 5‐point Likert scale was used, ranging from “strongly agree” to “strongly disagree.” In section 5 (4 questions), participants indicated their willingness to implement an IPEP as part of PE class (if they did not already do so). A 5‐point Likert scale was used to determine the factors influencing their willingness to implement an IPEP in PE class. How much time should be spent on an IPEP, and their opinion on where an IPEP should be included in post‐primary school was queried. Section 6 (3 questions) ascertained PE teachers' self‐perceived ability to implement an IPEP in a traditional PE class format. A 5‐point Likert scale was used to determine PE teachers' opinion on their readiness to implement an IPEP. An additional, open‐ended question was included enquiring on what factors and supports could help motivate them to implement an IPEP.

### Data Analysis

Responses were gathered from Qualtrics and imported into SPSS Statistics for Windows (version 25, IBM Corporation). Data were screened for omissions or invalid responses. Missing data was treated case by case. Frequencies and descriptive statistics were generated from eligible responses.

An attitude toward IPEPs scale was created by assigning a score for each available response to the 8 statements, ranging from a value of 1 being assigned to all “strongly disagree” responses and a value of 5 being assigned to all “strongly agree” responses. The scoring of negative statements was reversed to facilitate that a higher value on the scale equates to a more positive attitude toward IPEPs. Scores were compiled to give a maximum score of 40 (extremely positive attitude toward IPEPs). This process was repeated for the other key themes of the survey including, current understanding of IPEPs (min 3, max 15 points), willingness to implement an IPEP (min 5, max 25 points), and perceived ability to implement an IPEP (min 9, max 45 points). Data were non‐normally distributed and Mann‐Whitney *U* tests were used to identify significant differences between the following subgroups: gender, years teaching PE, teachers who have received formal IPEP education previously, teachers who are aware of specific IPEPs, school location, school socioeconomic status, teachers who currently use an IPEP in class, teachers who were willing to implement and IPEP in class, and teachers wishing to receive further IPEP education. Statistical significance was set at p < .05, with effect sizes classified at 0.1‐0.2 (small), 0.3‐0.4 (medium), 0.5 or greater (large).^24^

## RESULTS

A total of 336 responses were collected. After screening for eligibility (n = 2 ineligible) and completion (n = 47 insufficient), 287 were included for analysis. This represents 9.6% of registered PE teachers in Ireland. The demographic information for respondents is outlined in Table 1.

**Table 1:** Demographic Information for PE Teachers

Parameter n = 287	Value
PE teacher demographics	
Gender % (n)	
Male	46.00 (132)
Female	53.31 (153)
Prefer not to say	0.70 (2)
Total years teaching PE (mean ± SD)	11.24 ± 8.82
Year groups taught in previous 2 years % (n)	
First year	97.56 (280)
Second year	96.86 (278)
Third year	93.38 (268)
Fourth year	87.46 (251)
Fifth year	90.24 (259)
Sixth year	82.93 (238)
Teachers who have received formal education on IPEPs % (n)	
Received training	19.86 (57)
No training	80.14 (230)
Teachers who are aware of specific IPEPs % (n)	
Aware	29.97 (86)
Not aware	69.69 (200)
Type of student population % (n)	
Mixed	66.90 (192)
All male	14.98 (43)
All female	18.12 (52)
School location % (n)	
Urban	68.64 (197)
Rural	31.26 (90)
School DEIS status % (n)	
DEIS	22.66 (65)
Not DEIS	75.61 (217)
Unsure	1.74 (5)
School facilities available % (n)	
Indoor sports hall	90.94 (261)
Gym/fitness suite	52.96 (152)
Outdoor yard/hard surface	81.18 (233)
Outdoor pitch	76.66 (220)
Swimming pool	5.57 (16)
Other	6.27 (18)

DEIS, school in a disadvantaged community; IPEP, injury prevention exercise program.

### Current Use of IPEPs in PE Class

Only 19.4% (n = 54) of PE teachers reported to have used an IPEP in PE class within the previous 2 years. The data in the rest of this section relates to this 19.4% who used an IPEP. When these teachers were asked what encouraged them to use an IPEP, the 3 most frequent responses were “Adopted from a specific sport” (59.3%), “Awareness of injuries being common in students” (57.4%), and “current research shows its benefits” (38.9%). Most respondents (90.7%) reported using a self‐designed IPEP (44.4%) or a combination of a specific IPEP and self‐designed program (46.3%). Of those who used a specific IPEP, none reporting using it exactly as described, with all adapting components to fit their needs. The most common IPEPs reported were the GAA15 (n = 12) and FIFA11 (n = 6).

When asked about the components of the IPEP they use, the most cited elements were “Gentle running” (92.6%), “Flexibility” (88.9%), “Body weight exercises” (79.6%), “Jumping & landing technique” (77.8%), and “Agility” (76.0%). The least mentioned were “Heavy resistance training” (9.3%), and “Plyometrics” (44.4%). The most common duration of an IPEP in class was “5–10 minutes,” with 64.8% of respondents reporting this timeframe and 77.8% reporting spending less than 10 minutes on an IPEP. Most respondents implemented the program at the beginning of the class (74.1%).

### Attitudes Toward IPEPs

Of the 266 respondents who recorded attitude scores, 77.8% (n = 207) agreed that PE class is a suitable environment for implementing an IPEP. In addition, 91.4% agreed that it is important for PE teachers to have knowledge of IPEPs (n = 243), while 74.8% (n = 199) believed implementing an IPEP would reduce the number of injuries among students. Just 6.5% of respondents agreed that IPEPs are only suitable for those who participate in sport (n = 17). The majority of respondents felt IPEPs should be included as part of the PE curriculum in schools (85.5%, n = 219).

With a maximum possible “attitudes score” of 40, a mean of 31.08 ± 3.94 (n = 266) was calculated across respondents. Those who currently used an IPEP (median = 33.00, IQR = 31.00–35.00) displayed significantly higher attitudes toward IPEPs than those who did not (median = 31.00, IQR = 29.00–33.00), with a large effect size (p = .001, g = 0.52).

### Willingness to Implement IPEPs in PE Class

Two hundred and fifty‐six respondents reported their willingness to implement IPEPs. In total, 80.5% (n = 206) stated they would be willing to implement, or already do implement an IPEP as part of PE class. Only 5.5% (n = 14) agreed that it was the responsibility of other exercise professionals to implement an IPEP, while 18.8% (n = 48) agreed that their students would not be willing to complete an IPEP in class. Twenty‐three percent (n = 59) felt their PE class was not long enough to devote time to an IPEP, but only 6.3% (n = 16) were not willing to make changes to their current PE content used in class. Five to ten minutes was the most popular timeframe PE teachers were willing to spend on implementing an IPEP (52.5%, n = 135), while 23.0% (n = 59) were willing to dedicate 11‐15 minutes. Only 12.5% (n = 32) would be willing to spend longer than 15 minutes on an IPEP. The majority (90.0%, n = 230) of PE teachers reported being interested in receiving more education on PE class IPEPs.

With a maximum possible “willingness to implement an IPEP” score of 25, respondents scored a mean of 17.62 ± 2.49 (n = 256). There was no significant difference between those who currently implemented an IPEP and those who did not implement an IPEP. Those who were previously aware of an existing IPEP (median = 17.00, IQR = 15.75–19.00) were less willing to implement an IPEP than those who were unaware (median = 18.00, IQR = 16.50–19.00), with a medium effect size (p = .02, g = 0.29) (Table 2).

**Table 2:** Mean “Willingness to Implement IPEP Scores” Based on Teacher Demographic

	Willingness to Implement IPEP Scores (Max Score = 25) n = 256			
Parameter (n)	Median (IQR)	Mean ± SD	Significance Value	Effect Size (Hedges g)
Gender				
Male (123)	18.00 (16.00–19.00)	17.07 ± 2.44	p = .002*	0.43 (medium)
Female (132)	18.00 (16.00–20.00)	18.13 ± 2.45		
Total years teaching PE				
More than 11.24 (99)	17.00 (15.00–18.00)	18.01 ± 2.32	p = .045*	0.29 (small)
Less than 11.24 (157)	18.00 (15.00–19.00)	17.37 ± 2.57		
School location				
Urban (173)	18.00 (17.00–20.00)	17.88 ± 2.46	p = .02*	0.34 (medium)
Rural (83)	17.00 (16.00–18.00)	17.03 ± 2.48		
School socioeconomic status				
DEIS (56)	17.00 (15.75–20.00)	17.38 ± 2.73	p = .51	0.12 (small)
Not DEIS (195)	18.00 (16.00–19.00)	17.67 ± 2.44		
Received formal IPEP training				
Yes (55)	18.00 (16.00–19.00)	17.36 ± 2.56	p = .37	0.13 (small)
No (201)	18.00 (16.00–19.00)	17.69 ± 2.48		
Aware of existing IPEP				
Yes (81)	17.00 (15.75–19.00)	17.12 ± 2.76	p = .02*	0.29 (small)
No (174)	18.00 (16.50–19.00)	17.83 ± 2.33		
Currently using an IPEP in PE class			p = .39	0.25 (small)
Yes (49)	18.00 (17.00–19.00)	18.12 ± 2.30		
No (207)	18.00 (16.00–19.00)	17.50 ± 2.53		
Willing to implement an IPEP				
Yes (179)	18.00 (17.00–20.00)	18.00 (17.00–20.00)	p < .001*	2.42 (large)
No (9)	13.50 (11.25–14.75)	12.89 ± 2.37		
Would like to receive further education on IPEPs				
Yes (230)	18.00 (16.50–19.00)	17.83 ± 2.36	p < .001*	1.08 (large)
No (18)	15.50 (13.75–17.25)	15.28 ± 2.44		

* Statistical significance.

DEIS, School in a disadvantaged community; IPEP, Injury Prevention Exercise Program.

### Perceived Ability to Implement an IPEP

Of the 248 respondents who reported their perceived ability to implement an IPEP, 44.4% (n = 110) felt they had adequate skills to implement an IPEP, whereas 48.8% (n = 121) of respondents agreed that they lacked sufficient knowledge to implement an IPEP. Almost 3 in 4 teachers “agreed” or “strongly agreed” that they would be able to correct student's technique if they implemented an IPEP (73.4%, n = 182). Twenty‐one percent (n = 52) felt they lacked the facilities to implement an IPEP, while 33.5% (n = 83) “strongly disagreed” or “disagreed” that they had sufficient educational resources to assist them implementing an IPEP; 71.8% (n = 178) felt they would have the support from school management to implement an IPEP.

With a maximum possible “perceived ability to implement an IPEP” score of 45, respondents scored a mean of 29.8 ± 3.37 (n = 248). Those who currently implemented an IPEP (median = 35.00, IQR = 32.00–36.00) displayed a higher perceived ability to implement an IPEP than those who did not (median = 28.50, IQR = 25.00–32.00), with a large effect size (p < .001, g = 1.22). Those who received previous IPEP training (median = 34.00, IQR = 30.00–36.00) also displayed a higher score than those who did not (median = 29.00, IQR = 25.00–32.00), with a large effect size (p < .001, g = 0.86). This was the only measure which reported a significant difference between gender, with a significantly higher perceived ability to implement and IPEP in males (median = 31.00, IQR = 27.00–35.00) versus females (median = 29.00, IQR = 24.00–32.00), with a large effect size (p = .01, g = 0.33) (Table 3).

**Table 3:** Mean “Perceived Ability to Implement IPEP Scores” Based on Teacher Demographic

	Perceived Ability to Implement IPEPs Score (Max Score = 45) n = 248			
Parameter (n)	Median (IQR)	Mean ± SD	Significance Value	Effect Size (Hedges g)
Gender				
Male (119)	31.00 (27.00–35.00)	30.68 ± 5.19	p = .01*	0.33 (medium)
Female (128)	29.00 (24.00–32.00)	28.92 ± 5.43		
Total years teaching PE				
More than 11.24 (96)	28.00 (24.00–34.00)	30.31 ± 5.33	p = .23	0.16 (small)
Less than 11.24 (152)	29.00 (26.00–33.25)	29.46 ± 5.40		
School location				
Urban (166)	30.00 (26.00–33.50)	29.80 ± 5.46	p = .995	0.01 (small)
Rural (82)	30.00 (26.00–35.00)	29.77 ± 5.23		
School socioeconomic status				
DEIS (54)	28.50 (24.75–32.00)	28.43 ± 5.19	p = .03*	0.32 (medium)
Not DEIS (189)	30.00 (27.00–34.00)	30.11 ± 5.35		
Received formal IPEP training				
Yes (51)	34.00 (30.00–36.00)	33.27 ± 4.26	p < .001*	0.86 (large)
No (197)	29.00 (25.00–32.00)	28.89 ± 5.28		
Aware of existing IPEP				
Yes (78)	32.50 (28.00–36.00)	31.73 ± 5.26	p < .001*	0.57 (large)
No (169)	29.00 (26.00–32.00)	28.80 ± 5.07		
Currently using an IPEP in PE class				
Yes (48)	35.00 (32.00–36.00)	34.56 ± 3.85	p < .001*	1.22 (large)
No (200)	28.50 (25.00–32.00)	28.65 ± 5.06		
Willing to implement an IPEP				
Yes (173)	30.00 (27.00–33.50)	30.02 ± 4.83	p = .15	0.95 (large)
No (8)	26.50 (15.75–32.75)	25.25 ± 8.38		
Would like to receive further education on IPEPs				
Yes (230)	30.00 (26.00–35.00)	29.85 ± 5.28	p = .79	0.15 (small)
No (18)	28.50 (24.00–35.25)	29.06 ± 6.61		

* Statistical significance.

DEIS, School in a disadvantaged community; IPEP, Injury Prevention Exercise Program.

### Barriers and Facilitators to IPEP Implementation

When asked what other factors or supports would increase motivation to use an IPEP in class, some of the most frequently reported suggestions included “Training/CPD” (n = 41), “Educational resources/lesson plans” (n = 24), “Time constraints/timetabling issues” (n = 16) and “Student motivation” (n = 10).

## DISCUSSION

This study found that less than 1 in 5 PE teachers in Ireland currently use IPEPs in PE class. This is lower than the reported use in sports worldwide, such as youth soccer (30%)^23^ and collegiate women's soccer (66%),^25^ and within Ireland in sports such as camogie (34%),^22^ ladies football (48%).^26^ This is unsurprising as previous IPEP trends have largely targeted sports settings, while only recent research has encouraged the exploration of IPEPs in school environments.^13^ Despite the extensive focus on IPEPs within sport, most studies show less than half of sports teams currently use an IPEP. Only collegiate women's soccer has demonstrated a wide‐spread uptake, although the study suffered from a small sample size, with only 29 coaches completing the survey.^25^ This indicates that despite the discrepancy between IPEP research and promotion within these settings, the gap in usage is relatively small. This could imply that either sports settings are limited in their implementation capabilities, or that the school setting lends itself well to IPEP usage.

While current IPEP usage in schools is relatively low (19%), the majority of teachers (85.5%) reported IPEPs should be included in PE class and indicated they would like to learn more about how to do this. This suggests a discrepancy between PE teachers' positive attitudes toward IPEPs but relatively low IPEP implementation rates. This may be in part due to a perceived lack of ability to implement IPEPs resulting from a lack of training around the area, as less than 20% of respondents previously received formal IPEP education. Those who received previous IPEP training, were already aware of existing IPEP programs, and those who currently implemented IPEPs in class reported a significantly higher perceived ability to implement IPEPs than those who did not. In addition, “training/CPD” and “educational resources” were the 2 most reported factors which would encourage teachers to adopt an IPEP in class. The benefits of training and education on IPEP implementation is supported by a study on camogie coaches in Ireland, which demonstrated significant increases in perceived ability to implement an IPEP after a single 2 hours IPEP workshop.^27^ In fact, the use of an IPEP by coaches with their team increased from 15% pre‐workshop, to 73% 4 weeks post‐workshop.^27^ This increase in uptake was reasoned to be largely due to the multifactorial strategies adopted by the workshop, including formal training for coaches on both the theoretical and practical aspects of the program, easily accessible resources and materials, and formal endorsement at the national and/or regional sporting organizational level.^27^ However, it should be noted that the 4 week follow up in this study is relatively short, and longer‐term follow ups are necessary to establish if this increased uptake is maintained. In addition, of the 98 coaches who participated in the workshop, only 40 completed the 4‐week post‐workshop questionnaire, therefore there is a risk of confirmation bias associated with these findings. Despite this, the benefits of coach workshops on IPEP implementation well researched, with one‐off workshops demonstrating significant improvements in coach self‐efficacy,^28^ increased intent to implement IPEPs,^29^ and improved team adherence to the program.^30^ This suggests that delivering IPEP workshops are a useful tool for improving implementors perceived ability to deliver a program and can positively affect program implementation.

Most teachers who reported using an IPEP in class used a self‐designed, or a combination of self‐designed and an existing sport‐specific IPEP. This indicates that no existing appropriate programs are available for teachers to utilize, or that teachers are not aware of any to implement. Therefore, for a teacher to implement an IPEP in PE class, it may require additional work for teachers to create the program. Additionally, there is a risk of potentially inappropriate exercises being included, or usage of existing sport‐specific IPEPs, which may not be ideal for the general adolescent population. These may be contributing factors to the low usage of IPEPs among teachers. This is supported in the results as “training/CPD” was reported to be the biggest factor which would increase teachers' motivation to implement an IPEP. In fact, those already aware of existing IPEPs were significantly less willing to implement an IPEP than those who were unaware of existing IPEPs. It may be that teachers are aware they are not suitable for PE class, and thus less willing to implement them. When asked to name the existing IPEP they knew of, no school‐specific IPEP was named, with the sport‐specific GAA15 (n = 12) and FIFA11 (n = 6), the most frequently reported IPEPs. The importance of a suitable IPEP for the target population has already been highlighted in football, as the lack of a link to football‐related goals and lack of participant enjoyment and engagement was reported as key barriers to program implementation.^17^ Meanwhile, research into implementation of interventions in school settings has suggested the lack of congruency between the programs' aims and principles and the implementors beliefs will adversely affect implementation.^21^ Furthermore, the perceived benefits of an intervention was frequently reported as a barrier to school‐based health interventions,^31^ suggesting students performing an IPEP designed for a sport which they do not participate in may reduce compliance with the program if they do not feel like it is relatable for them. If teachers do not see the relationship between the IPEP and their students, they may be less likely to implement the program. As such, the development of a suitable school‐specific IPEP may improve willingness to implement an IPEP in this population.

Barriers are factors which impede program implementation and compromise program fidelity.^18^ Just over 1 in 5 PE teachers reported a lack of facilities and insufficient time as barriers to implementing an IPEP in PE class, which are also well established barriers to IPEP implementation in sports settings.^17^, ^32^ To ensure the program is delivered as intended (program fidelity), addressing these barriers is essential. PE teachers would like to spend between 5 and 15 minutes per PE class on an IPEP. Despite the vast majority having access to an indoor hall, not all PE classes will take place there, with class often taking place on courts or pitches. To account for this, programs should be adaptable, and capable of being performed in variety of different settings and conditions.^18^, ^19^, ^21^, ^33^ Thus, ensuring IPEPs in a PE class context require minimal equipment, are of short duration and can be completed in different settings appears to be important for successful implementation. This may highlight some of the misconceptions surrounding IPEP accessibility as many existing sport specific IPEPs (eg, FIFA11+, GAA15) already fit this profile.^34^, ^35^ However, as these program contents are sport‐specific and designed for a relatively homogenous population, their suitability for a more heterogenous population is questionable. Thus, improving PE teachers' knowledge on IPEPs may address some of the perceived barriers to implementation.

One of the biggest opportunities for a successful and sustainable school‐based IPEP appears to be PE teachers' attitudes toward IPEPs. The vast majority of teachers felt IPEPs should be included as part of the PE curriculum, while over 3 in 4 agreed that PE class is a suitable environment for an IPEP, and that implementing one would reduce the number of injuries among students. The attitudes of PE teachers is a crucial factor in potential implementation of a PE‐based IPEP as previous research has shown the implementors characteristics to be a key factor in sustaining school health interventions.^31^ In a sporting context, youth football coaches believe their attitudes toward IPEPs influenced their players motivation to perform the program, while there was substantially lower compliance among players if their coach felt the IPEP was too time consuming, or did not have enough football‐specific activities.^36^ Considering the lack of formal IPEP education reportedly received by PE teachers, their existing positive attitudes toward IPEPs is advantageous for implementation and likely stems from their expertise in the related fields of physical education and physical activity.

### Limitations

This survey focused on the opinions and perceptions of post‐primary PE teachers. Despite being key stakeholders in any PE class intervention, other key stakeholders like school management and students could inform a more comprehensive understanding of IPEPs within PE class. Additionally, to capture the most current IPEP practices, only those who implemented an IPEP in class within the previous 2 academic years were asked to provide information regarding their practices. It is possible that the global COVID‐19 pandemic, which disrupted academic schedules during the collection period, may have influenced the findings. In addition, this study used a research designed survey, a validated measure may have enable a more robust survey, however, to the authors knowledge, no such measure currently exists. Lastly, for survey distribution, schools were emailed as opposed to teachers directly for data protection reasons. With no way of knowing how many teachers received the survey, the response rate could not be determined by this method. In lieu of this, response rate was calculated based on the maximum number of possible respondents.

### Conclusion

While PE teachers have demonstrated a willingness to implement IPEPs, most currently lack the training and resources required to carry out a program. This study also suggests there is a need for a school‐specific IPEP to ensure an effective and sustainable program.

## IMPLICATIONS FOR SCHOOL HEALTH

The majority of PE teachers feel PE class is a suitable environment for IPEPs and believe it would reduce the number of injuries sustained by adolescents. This suggests post‐primary PE class may be an under‐utilized setting for adolescent injury prevention and should be further investigated. Schools should explore the potential for its inclusion as preventing injuries among adolescent populations can have major implications from a physical,^5^ psychological,^7^ and financial^8^, ^9^ perspective. IPEPs which require little financial or time resources already exist in sports settings,^34^, ^35^ highlighting the potential for implementation in even the most resource restricted schools. However, contents should be tailored to suit the general student population and must be adaptable to cater for such a heterogeneous population.

### Human Subjects Approval Statement

Ethical approval was granted by the Dublin City University Human Research Ethics Committee (Reference number DCUREC/2020/255). Informed consent was gathered prior to commencing the survey.

### Conflict of Interest

There are no financial or nonfinancial conflicts of interest to disclose from any of the named authors who contributed to this study.
